# Serum concentrations of antidepressants, antipsychotics, and antiepileptics over the bariatric surgery procedure

**DOI:** 10.1007/s00228-021-03182-1

**Published:** 2021-07-16

**Authors:** Susanna M. Wallerstedt, Karin Nylén, Magnus A. B. Axelsson

**Affiliations:** 1 Department of Pharmacology, Sahlgrenska Academy, University of Gothenburg, Gothenburg, Sweden; 2 HTA-Centrum, Sahlgrenska University Hospital, Gothenburg, Sweden; 3 Department of Clinical Chemistry, Sahlgrenska University Hospital, Gothenburg, Sweden

**Keywords:** Antidepressants, Antiepileptics, Antipsychotics, Bariatric surgery, Pharmacotherapy, Therapeutic drug monitoring

## Abstract

**Purpose:**

As a substantial proportion of bariatric surgery patients use psychotropic/antiepileptic drugs, we investigated the impact of this procedure on serum concentrations.

**Methods:**

In a naturalistic, longitudinal, prospective case series, we compared dose-adjusted trough concentrations of antidepressants, antipsychotics, or antiepileptics in consecutive patients before and after bariatric surgery. Adherence to treatment over 2 weeks preceding each sampling was considered.

**Results:**

In all, 85 participants were included (86% female, median age 45 years, median body mass index 42 kg/m^2^). They were being treated with 18 different psychotropic/antiepileptic drugs (7 substances: 6–17 individuals, 11 substances: 1–4 individuals) and contributed 237 samples over a median of 379 days after surgery. For four out of seven substances with pre-/post-surgery samples available from six or more individuals, the dose-adjusted concentration was reduced (sertraline: 51%, mirtazapine: 41%, duloxetine: 35%, citalopram: 19%). For sertraline and mirtazapine, the low-calorie-diet before surgery entirely explained this reduction. A consistent finding, irrespective of drug, was the association between the mean ratio of the post-/pre-diet dose-adjusted concentration and the lipophilicity of the drug (logD; correlation coefficient: −0.69, *P* = 0.0005), the low-calorie diet often affecting serum concentration more than the surgery itself.

**Conclusions:**

Serum concentrations of psychotropic/antiepileptic drugs vary after bariatric surgery and can be hard to predict in individual patients, suggesting that therapeutic drug monitoring is of value. Conversely, effects of the pre-surgery, low-calorie diet appear generalizable, with decreased concentrations of highly lipophilic drugs and increased concentrations of highly hydrophilic drugs. Interaction effects (surgery/dose/concentration) were not evident but cannot be excluded.

## Introduction

Every year, several hundreds of thousands of individuals worldwide undergo bariatric surgery [1]. According to a recent systematic review, this intervention may affect drug bioavailability, but, so far, too little is known to make dosing recommendations [2].

Psychiatric comorbidity is common among bariatric surgery candidates [3, 4], and most patients continue their use of psychiatric medications after surgery [5]. However, there is a scarcity of data concerning potential consequences of bariatric surgery regarding the concentration of drugs used for these conditions [6]. Such knowledge is essential because reduced concentrations may contribute to relapses and increased concentrations could lead to adverse effects; also, in psychiatry, therapeutic effects are not as easy to monitor as, for instance, blood pressure or blood glucose. As far as we are aware, there are only two longitudinal studies investigating patients on treatment, both severely restricted in terms of the number of substances investigated and the number of patients per substance [7, 8]. Cross-sectional studies, on the other hand, have been limited to the two antidepressants sertraline and duloxetine, and this design cannot provide knowledge on causality [9, 10]. Although scarce, the available literature suggests that there may be non-negligible changes in psychotropic drug concentrations after bariatric surgery. Therefore, we aimed to investigate serum concentrations of antidepressant, antipsychotic, and antiepileptic drugs in a naturalistic setting over the bariatric surgery procedure.

## Methods

We designed the study as a naturalistic longitudinal, prospective case series with intra-individual comparisons of drug concentrations over time, before and up to 1 year after the surgical procedure.

Between September 2013 and May 2016, we screened all patients referred for bariatric surgery in Region Västra Götaland, Sweden, regarding treatment with psychotropic and/or antiepileptic drugs. Consecutive patients, treated with such drugs and planned for surgery at Sahlgrenska University Hospital, were asked to participate. Patients planned for surgery in other hospitals were not eligible. Inclusion criteria were current treatment with an antidepressant, antipsychotic, or antiepileptic drug; Swedish-speaking patients; and written informed consent. Exclusion criteria included that bariatric surgery, in the end, was not performed or performed in another hospital, or that the psychotropic/antiepileptic medication had been withdrawn or was restricted to agomelatine, a substance with too short a half-life for a meaningful concentration analysis.

The participants contributed blood samples to the current study during routine pre-surgery visits before, and post-surgery visits about 8 weeks, 6 months, and 1 year after, the surgery. Pre-surgery sampling generally occurred twice: at an initial preoperative assessment visit, and just before surgery. Between these visits, that is, before surgery, patients were required to adhere to a low-calorie diet and show a weight reduction. The samples were stored as serum in −80 °C at a biobank, and analysed after all samples had been collected.

Before every routine visit at which the sampling was to occur, we contacted the participants by telephone to systematically obtain information on the current dose as well as adherence to the drug treatment in question over the 2 preceding weeks. Samples were excluded from the data set if non-adherence less than four elimination half-lives prior to sampling was reported; exceptions were made for occasional missed doses of fluoxetine. Half-lives applied were those described for a drug or its metabolite in the summary of product characteristics. We also asked for and recorded concomitant drug treatment, weight, and smoking status. To be able to relate potential changes in concentration to health consequences, we asked the participants during the follow-up phone calls, using an open question, if anything had happened to their health since the last visit. To obtain trough blood concentrations, we strived to schedule the routine visits before lunchtime and the participants were instructed not to take morning tablets before the sampling.

Specific substances were analysed concomitantly, using clinical routine methods, at accredited laboratories at the Department of Clinical Chemistry, Sahlgrenska University Hospital, Gothenburg, Sweden, or (as for gabapentin, hydroxybupropion, mianserine, pregabalin, paroxetine and topiramate) at the Department of Clinical Pharmacology, St. Olav’s Hospital, Trondheim, Norway. Analysis methods were based on liquid chromatography/tandem mass spectrometry or (for lamotrigine and valproic acid) enzyme immunoassay. For fluoxetine, venlafaxine, amitriptyline, and clomipramine, both the parent and the metabolite were analysed; for bupropion, the metabolite hydroxybupropion was analysed. The lower limit of quantification was well below measured values for all samples eligible for inclusion. Measured total coefficients of variation (at analysis ± 6 months) were ≤ 5.2% at all internal control levels for in house liquid chromatography/tandem mass spectrometry methods. Enzyme immunoassays were compliant with expected total coefficients of variation ≤ 6.1%. As for trueness, all analyses were compliant with relevant external control programs.

Statistical analyses were performed using SPSS, version 23.0 (IBM SPSS Statistics for Windows, Armonk, NY, USA). Participants treated with the same substance before and after surgery, with one or more trough sample concentrations available, were included in the analysis. To allow interindividual and intraindividual comparisons, serum concentrations were adjusted for defined daily dose (DDD) [11]. For example, since DDD for citalopram is 20 mg, a DDD-adjusted citalopram concentration of 100 nmol/L could represent a measured concentration of 50, 100, or 200 nmol/L, given a daily dose of 10, 20, or 40 mg, respectively. To compare pre- to post-surgery concentrations, as well as pre- to post-diet concentrations, we used Wilcoxon signed rank test for substances with samples available for six or more individuals. We used Spearman’s correlation to explore the relationship between the ratio of the post-/pre-diet concentrations and (i) the distribution coefficient for the drug in question, i.e., the logarithm of the ratio of its concentration in a lipophilic and a hydrophilic solution (logD), as well as (ii) the weight loss in kilograms for pregabalin.

## Results

**Fig. 1:**
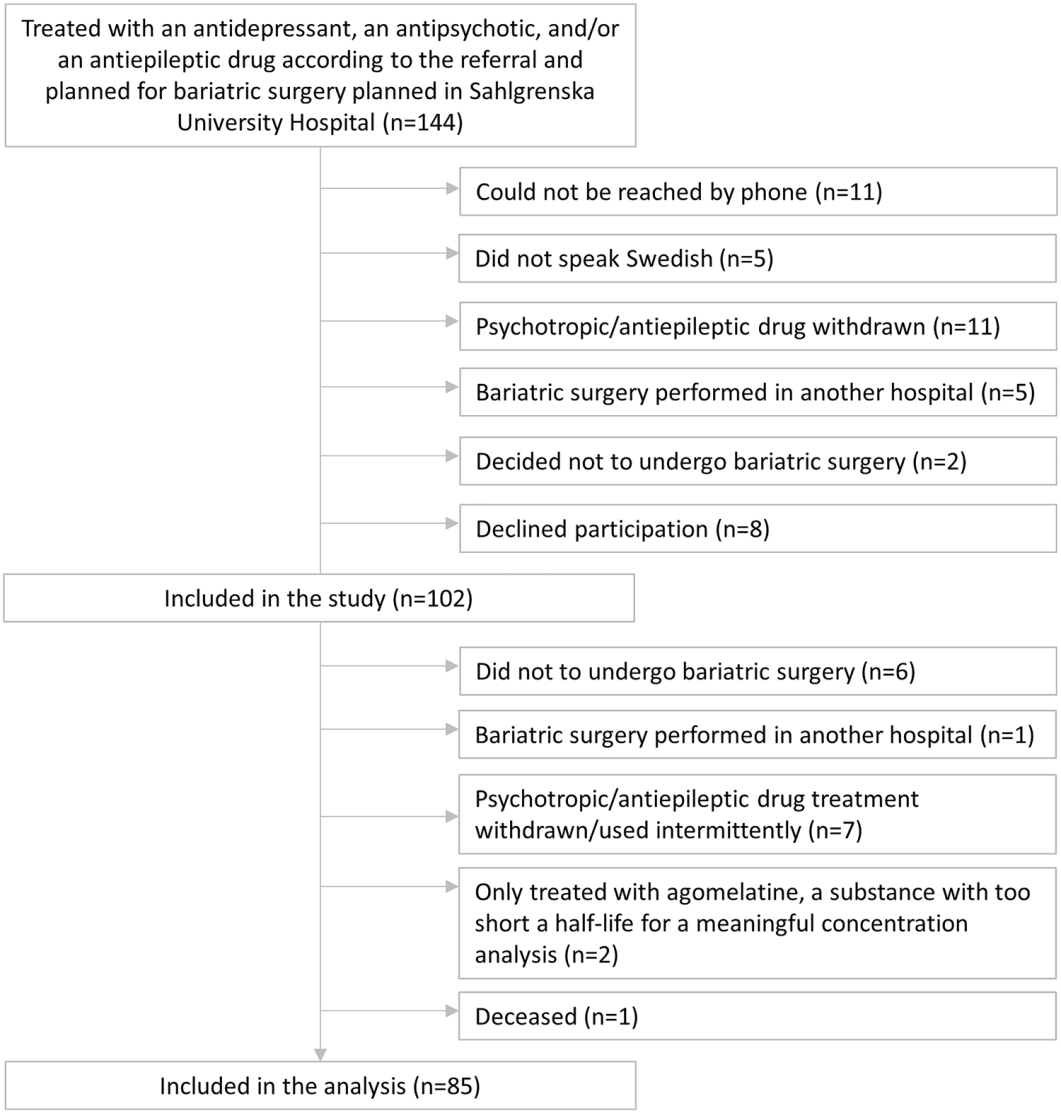
Participant flowchart

A total of 1,950 patients were screened, 429 (22%) of whom were being treated with a psychotropic and/or an antiepileptic drug. Out of 144 consecutive patients who (i) were prescribed one or more psychotropic/antiepileptic drugs, according to the referral, and (ii) were planned for surgery at Sahlgrenska University Hospital, 85 were included in the present analysis (Fig. 1; Table 1).

**Table 1:** Baseline characteristics of patients included in the study (*n* = 85)

Age, yrs			45 (38–54)
Female sex			73 (86)
BMI, kg/m^2^			42 (40–45)
Smoking			15 (18)
Number of regular prescription drugs^a^			4 (2–6)
Psychotropic/antiepileptic substance	SSRIs	Citalopram/escitalopram	18 (21)
		Fluoxetine	15 (18)
		Paroxetine	1 (1.2)
		Sertraline	13 (15)
	SNRIs	Duloxetine	9 (11)
		Venlafaxine	16 (19)
	TCAs	Amitriptyline	3 (3.5)
		Clomipramine	2 (2.4)
	Other antidepressants	Bupropion	2 (2.4)
		Mianserine	3 (3.5)
		Mirtazapine	6 (7.1)
	Antipsychotics	Olanzapine	1 (1.2)
		Quetiapine	2 (2.4)
	Antiepileptics	Gabapentin	2 (2.4)
		Lamotrigine	4 (4.7)
		Pregabalin	7 (8.2)
		Topiramate	4 (4.7)
		Valproic acid	2 (2.4)
Reason for treatment^b^	Depression		50 (59)
	Anxiety		22 (26)
	Pain		9 (11)
	Fibromyalgia		5 (5.9)
	Bipolar disease		5 (5.9)
	Burnout		4 (4.7)
	Sleep problems		2 (2.4)
	Epilepsy		1 (1.2)
	Other		10 (12)
Surgery	Gastric bypass		67 (79)
	Sleeve		18 (21)

Values are presented as medians (interquartile range (IQR)) or n (percentage)

*BMI* body mass index, *SNRI* serotonin–norepinephrine reuptake inhibitor, *SSRI* selective serotonin reuptake inhibitor, *TCA* tricyclic antidepressant

^a^Reflecting burden of disease (see Brilleman and Salisbury [31])

^b^Some patients had more than one reason for treatment with a psychotropic/antiepileptic drug

**Fig. 2:**
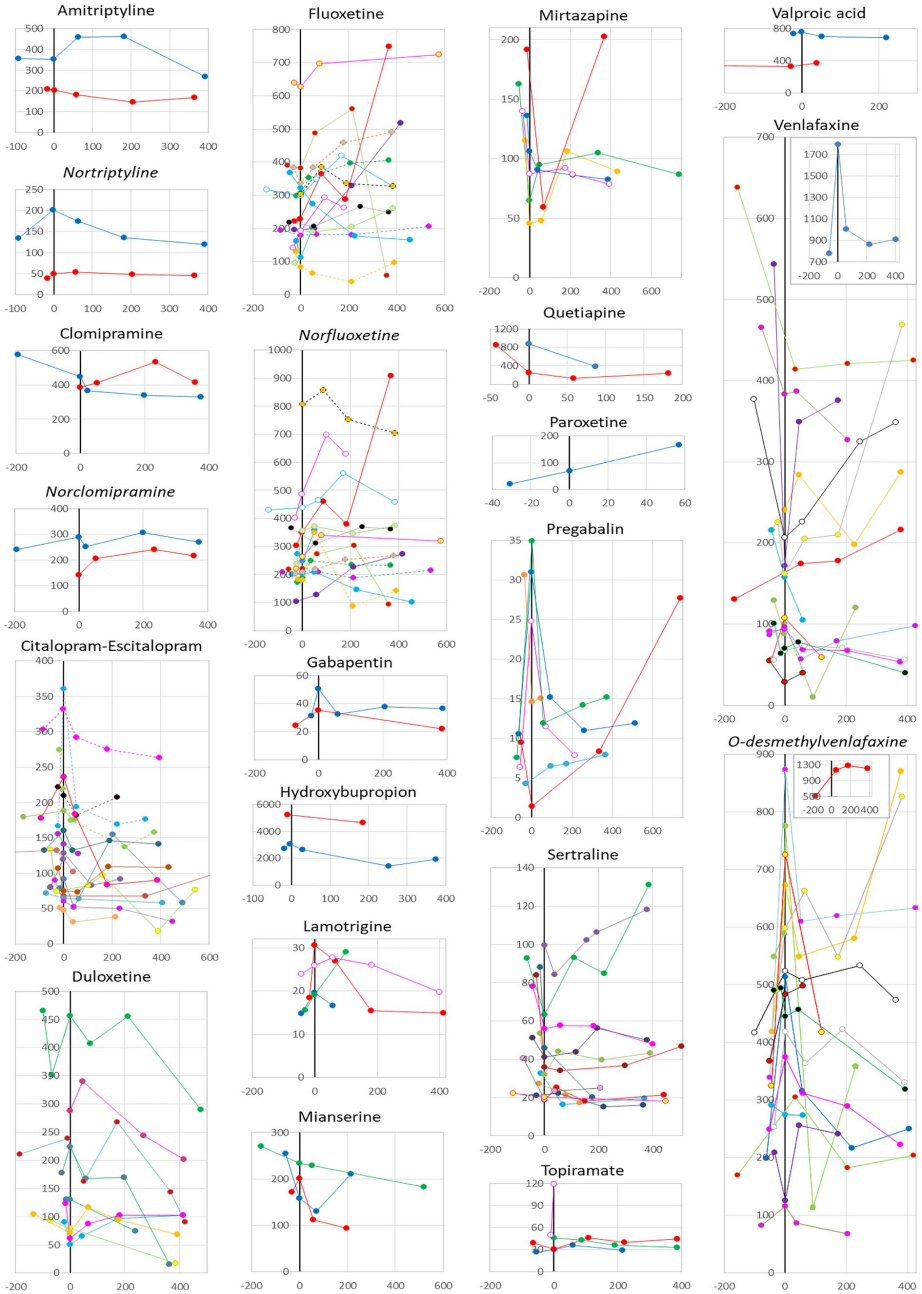
Dose-adjusted steady-state trough serum concentrations, plotted per patient against days before and after bariatric surgery. Values are expressed as serum concentrations (µmol/L for antiepileptics, nmol/L for other substances), multiplied by the defined daily dose (DDD) and divided by the current daily dose (Y axis), plotted per patient against days before and after bariatric surgery (X axis). For citalopram/escitalopram, dashed lines depict continuous concomitant use of omeprazole. Metabolites are shown immediately after the parental compound and labelled in italics (not applicable for hydroxybupropion since bupropion was not analysed); individual patients are shown with the same colour/pattern for parental compound and metabolite

The participants were being treated with a selective serotonin reuptake inhibitor (SSRI) (*n* = 47), a serotonin–norepinephrine reuptake inhibitor (SNRI) (*n* = 25), a tricyclic antidepressant (TCA) (*n* = 5), another antidepressant (*n* = 11), an antipsychotic drug (*n* = 3), and/or an antiepileptic drug (*n* = 19). Drug concentrations before and after bariatric surgery were obtained for 18 substances, with one to 17 individuals for each substance (Table 2). In median, the last sampling occurred 379 days after surgery (interquartile range (IQR) 214–406), when the body mass index (BMI) had been reduced from 42 (40–45) to 30 (27–33) kg/m^2^. Plotting the dose-adjusted concentration over time for each individual, we found large variations between individuals (Fig. 2). For four out of seven substances with pre- and post-surgery samples available from six or more participants, the dose-adjusted concentration on the last day of sampling was reduced (sertraline: 51%, mirtazapine: 41%, duloxetine: 35%, citalopram: 19%). In retrospect, four participants reported drug-related psychiatric health aspects that were temporarily associated with a substantial (doubled/halved) change in dose-adjusted drug concentration (Table 3).

**Table 2:** Drug concentration pre-diet/pre-surgery and post-surgery (A), as well as pre-diet and post-diet (B)

		Individuals *N*	Dose-adjusted drug concentration^a^ Median (IQR)		
			Pre-diet/pre-surgery	Post-surgery^a^	*P*-value
A					
SSRI	Citalopram/escitalopram	17	133 (80–179)	108 (68–167)	**0.015**
	Fluoxetine	14	221 (157–371)	294 (193–570)	0.084
	Paroxetine	1	22 (N/A)	167 (N/A)	N/A
	Sertraline	11	51 (27–84)	25 (18–48)	**0.041**
SNRI	Duloxetine	8	127 (96–203)	82 (30–103)	**0.015**
	Venlafaxine	16	173 (88–443)	168 (57–369)	0.47
TCA	Amitriptyline	2	283 (N/A)	218 (N/A)	N/A
	Clomipramine	1	578	329	N/A
Other	Hydroxybupropion	2	3,997 (N/A)	3,298 (N/A)	N/A
	Mianserine	3	255 (N/A)	183 (N/A)	N/A
	Mirtazapine	6	148 (131–170)	88 (82–143)	**0.046**
Antipsychotics	Olanzapine	1	113 (N/A)	106 (N/A)	N/A
	Quetiapine	1	848 (N/A)	239 (N/A)	N/A
Antiepileptics	Gabapentin	2	34 (N/A)	35 (N/A)	N/A
	Lamotrigine	4	17 (15–23)	18 (15–27)	N/A
	Pregabalin	6	8.5 (5.8–16)	13 (8–18)	0.25
	Topiramate	2	33 (N/A)	37 (N/A)	N/A
	Valproic acid	2	533 (N/A)	529 (N/A)	N/A
B					
SSRI	Citalopram/escitalopram	17	133 (80–179)	124 (71–215)	0.87
	Fluoxetine	12	259 (147–379)	263 (182–333)	0.31
	Paroxetine	1	22 (N/A)	69 (N/A)	N/A
	Sertraline	8	66 (43–87)	39 (23–53)	**0.012**
SNRI	Duloxetine	8	127 (96–203)	105 (64–235)	0.21
	Venlafaxine	14	172 (79–399)	132 (87–215)	0.20
TCA	Amitriptyline	2	283 (N/A)	279 (N/A)	N/A
	Clomipramine	1	578 (N/A)	449 (N/A)	N/A
Other	Hydroxybupropion	1	2,732 (N/A)	3,067 (N/A)	N/A
	Mianserine	3	255 (N/A)	201 (N/A)	N/A
	Mirtazapine	4	138 (120–157)	76 (50–102)	N/A
Antipsychotics	Olanzapine	0			
	Quetiapine	1	848 (N/A)	260 (N/A)	N/A
Antiepileptics	Gabapentin	2	28 (N/A)	43 (N/A)	N/A
	Lamotrigine	4	17 (15–25)	23 (19–29)	N/A
	Pregabalin	5	9.5 (7–21)	25 (8–33)	N/A
	Topiramate	3	39 (N/A)	31 (N/A)	N/A
	Valproic acid	1	737 (N/A)	759 (N/A)	N/A

Significant P-values are bolded

*DDD* defined daily dose, *IQR* interquartile range, *N/A* not applicable, *SNRI* serotonin–norepinephrine reuptake inhibitor, *SSRI* selective serotonin reuptake inhibitor, *TCA* tricyclic antidepressant

^a^µmol/L for antiepileptics and nmol/L for other substances, the concentrations adjusted for dose in DDDs (see WHO Collaborating Centre for Drug Statistics Methodology [11]), with statistical comparisons (pre versus post) performed when n ≥ 6

^b^Last observation carried forward, at a median 379 (IQR 214–406) days after surgery

**Table 3:** Details regarding participants reporting drug-related psychiatric health aspects over the bariatric surgery procedure that, in retrospect, were temporarily associated with a substantial* change in the dose-adjusted drug concentration

Case No	Surgical procedure	Substance	Baseline		Post-surgery			Health consequences related to changed drug concentrations
			Drug dose	Concentration	Day	Drug dose	Concentration	
1	GBP	Citalopram	10 mg	137 nmol/L	+252	10 mg	69 nmol/L	Reported increased dose 3–4 weeks before the final sampling because of psychiatric symptoms
					+374	20 mg	158 nmol/L	
2	GBP	Citalopram	10 mg	89 nmol/l	+178	10 mg	42 nmol/L	Reported increased dose 2 weeks before the final sampling because of depressive symptoms
					+386	20 mg	90 nmol/L	
3	GBP	Mirtazapine	15 mg	96 nmol/L	+68	15 mg	30 nmol/L	Reported that sertraline had been switched to escitalopram after the first post-surgery sampling without beneficial effects, changed back to sertraline
					+368	30 mg	203 nmol/L	
		Sertraline	200 mg	130 nmol/L	+68	200 mg	66 nmol/L	
					+368	200 mg	78 nmol/L	
4	Sleeve	Mirtazapine	30 mg	140 nmol/L	213	30 mg	87 nmol/L	Reported increased dose 2 months before the final sampling because of the psychiatric condition
					393	45 mg	119 nmol/L	

*GBP* gastric bypass

^*^Doubled/halved

**Fig. 3:**
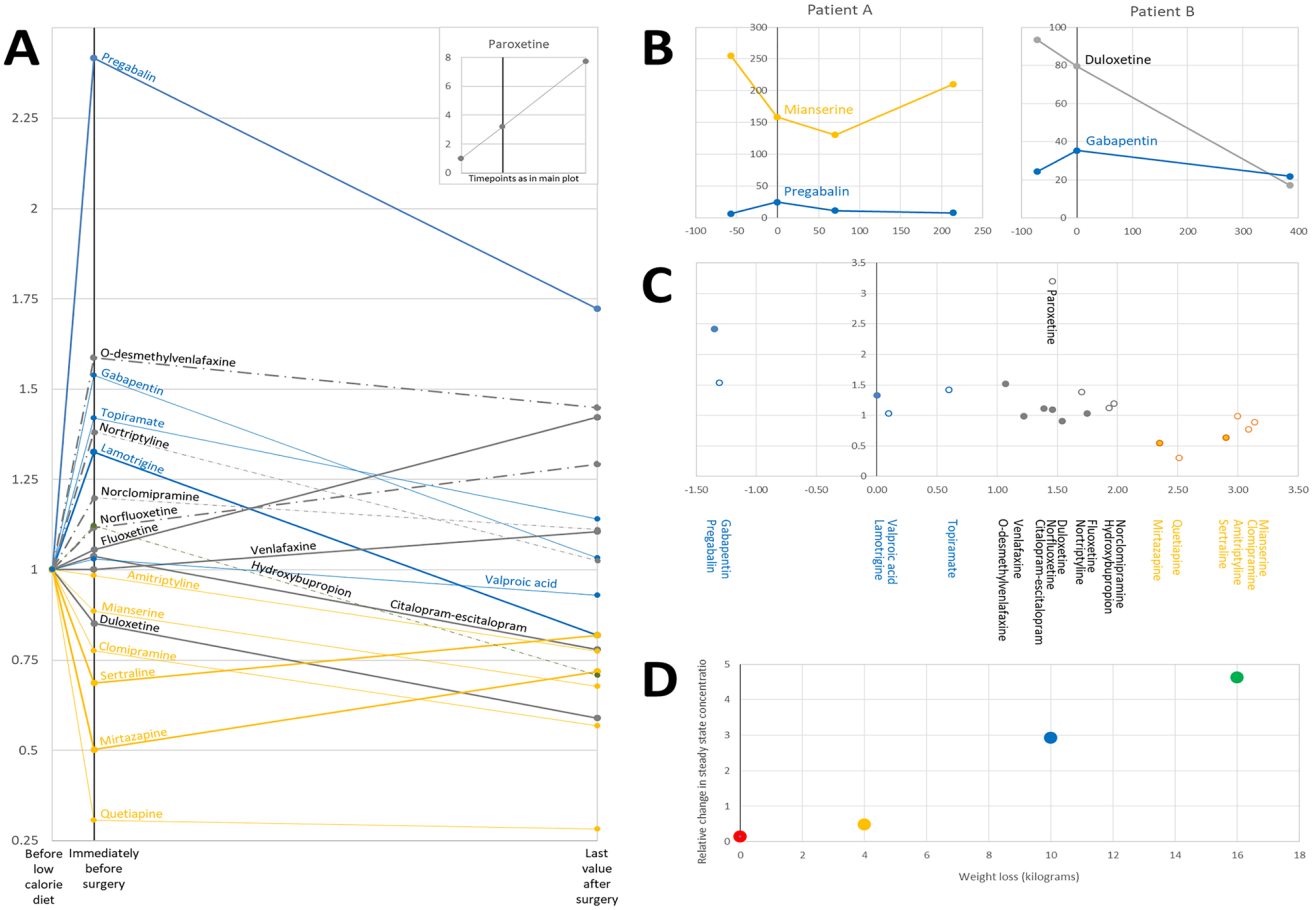
Relative change in dose-adjusted steady state concentration for highly hydrophilic substances (blue, logD at pH 7.4: < 1) and highly lipophilic substances (yellow, logD at pH 7.4: > 2) over the low-calorie diet and post-surgery periods (**A**), within the same patient (**B**), and over the low-calorie diet period (**C**), and for the highly hydrophilic substance pregabalin over the low-calorie diet period plotted against the weight loss (**D**). In **A**, values are expressed as the arithmetic means of the immediate (0–3 day) pre-surgery values (normally at the very end of the low-calorie diet), and 1-year (≥ 300 day) post-surgery values, respectively; both first divided by the baseline (pre-low-calorie diet) value for the particular patient (dashed lines = metabolites; thin lines = *n* < 4; bold lines = *n* ≥ 4). For paroxetine, quetiapine, and valproic acid, post-surgery concentrations at ≥ 300 days were not available. For these patients, the last post-surgery observation at 57, 182, and 220 days, respectively, was carried forward. In **B**, data from Fig. 2 from two patients (**A** and **B**) are merged to illustrate that hydrophilic and lipophilic drugs are affected differently by a low-calorie diet within the same patient (X axis, days before and after surgery; Y axis, nmol/L (mianserine, duloxetine) or µmol/L (pregabalin, gabapentin). In **C**, values are expressed as the arithmetic mean of immediate pre-surgery (normally sampled at the very end of the low-calorie diet) values, divided by their corresponding baseline (pre-low-calorie diet) values, plotted against the logD value of the drug at pH 7.4. In **D**, values are expressed as immediate pre-surgery (normally at the very end of low-calorie diet) values divided by their corresponding baseline (pre-low-calorie diet) values, and individuals are shown in same colours as in Fig. 2

As a change in dose-adjusted concentration occurred already after the pre-surgery diet, before the bariatric surgery and after a median weight reduction of 6 kg (IQR 5–8 kg), we plotted the relative change in serum concentration over time for all analysed parent substances and metabolites (Fig. 3A). We found that the dose-adjusted concentrations had increased for the most hydrophilic drugs (pregabalin, gabapentin, and lamotrigine), varied for the modestly more lipophilic ones (duloxetine and citalopram/escitalopram), and had decreased for the strongly lipophilic drugs (quetiapine, mirtazapine, and sertraline). A similar phenomenon was found when comparing venlafaxine to its more hydrophilic metabolite O-desmethylvenlafaxine, and could also be observed when a lipophilic and a hydrophilic drug were used concurrently by the same patient, i.e., when individual patient characteristics were constant (Fig. 3B). For sertraline, the only highly lipophilic/hydrophilic substance where a statistical comparison could be made, the dose-adjusted concentration was significantly reduced over the pre-surgery diet period (Table 2). After surgery, the effects of the low-calorie diet generally appeared to change direction for most drugs, and the dose-adjusted concentrations recovered towards baseline values. Exceptions were fluoxetine and its metabolite, norfluoxetine, which continued to increase also after surgery, and duloxetine, which continued to decrease.

As there seemed to be an association between the lipophilicy/hydrophilicity of a drug and the change in dose-adjusted concentration over the pre-surgery, low-calorie diet period, we plotted the distribution coefficient for each substance (logD) against the ratio between the post- and pre-diet concentrations (Fig. 3C). The logD value is a measure of the lipophilicity of a substance, defined as the logarithm of the partitioning ratio between octanol and water, meaning that, in a two-phase system, for example pregabalin (logD =  −1.35) would be > 20-fold more abundant in water than in octanol, and mianserine (logD = 3.14) would be more than 1 000-fold more abundant in octanol than in water (at pH 7.4). A post hoc analysis at the overall sample level, including all samples with a unique substance concentration available pre- and post-diet, gave a correlation coefficient of −0.42 between the ratio post-/pre-concentration and logD (*P* < 0.0001). At the substance level, correlating the mean of the ratio post-/pre-concentration for each substance to logD revealed a correlation coefficient of −0.69 (*P* = 0.0005).

Except for pregabalin, where the extent of weight loss covaried with the change in dose-adjusted concentration over the pre-surgery, low-calorie diet period (Fig. 3D; correlation coefficient: 1, *P* < 0.01), no patient characteristic, such as age, sex, weight loss, change in smoking status, or surgical technique (gastric bypass versus sleeve), was visually found to covary with serum concentrations (data not shown). Except for continuous omeprazole treatment, which was an exclusive feature of the four patients with the highest average citalopram serum concentration, probably illustrating the established CYP2C19-based pharmacokinetic interaction [12] (Fig. 2, dashed lines), no covariation was found that could be explained by concomitant medications (data not shown).

## Discussion

In this study, we show that reduced concentrations after bariatric surgery can be expected for citalopram, sertraline, duloxetine, and mirtazapine. Occasionally, a substantial decline in serum concentration was temporarily associated with a treatment adjustment in routine care, either a dose increase or a switch, which indirectly suggests that the patient/physician had observed a reduced effect. For sertraline and mirtazapine, reduced dose-adjusted serum concentrations were seen already after the pre-surgery, low-calorie diet period. The post-surgery reduction in sertraline concentration that was previously reported in a cross-sectional study [9] may therefore be attributed to the pre-surgery diet rather than the surgery itself. As far as we are aware, this course of events has not previously been shown. In previous longitudinal studies, the sampling was performed on one pre-surgery occasion [7, 8], and therefore did not include samples before and after any potential diet.

The inverse relationship between logD and the post-diet change in dose-adjusted drug concentrations suggests that the direction of this change may be determined by lipophilicity, with increased concentrations of very hydrophilic substances and reduced concentrations of very lipophilic substances. Underlying mechanisms for this can only be speculated on. Possibly, logD could be a confounder regarding the elimination route. The most hydrophilic drugs pregabalin and gabapentin are cleared by glomerular filtration with limited tubular reabsorption [13], and the decreased elimination after a low-calorie diet may be associated with a decreased obesity-related glomerular hyperfiltration. Indeed, 1 month of low-calorie diet has been reported to improve glomerular function proportionally to weight loss [14]. This appears compliant with our data demonstrating a correlation between the weight loss and the increase in the pregabalin concentration over the low-calorie diet. The less hydrophilic substances topiramate, O-desmethylvenlafaxine, and lamotrigine are also cleared renally, but after varying degrees of glucuronidation. Their increased dose-adjusted concentration after the low-calorie diet could be expected because glucuronidation increases in obesity [15, 16], and may therefore decrease after a low-calorie diet. By contrast, phase I metabolism in general, and cytochrome P450 (CYP)-mediated metabolism in particular, may increase following a low-calorie diet because of decreased liver inflammation and steatosis [2, 16, 17]. This may explain why the dose-adjusted concentration of the most cytochrome P450 3A4 (CYP3A4)-dependent substances, mirtazapine and sertraline, dropped conspicuously during the low-calorie diet, whereas the remaining substances, which are dependent on various phase I enzymes, showed varying and more modest changes.

For citalopram and duloxetine, with a logD of 1.39 and 1.54, respectively, the pre-surgery diet was not associated with reduced post-surgery concentrations, and the reduced dose-adjusted concentrations may therefore have been caused by the surgery itself, as previously suggested in two small longitudinal studies that included four patients on either citalopram [7] or escitalopram [8], and in a cross-sectional study investigating a duloxetine single dose ingestion [10]. Our results suggest that this surgical consequence may be of clinical relevance in some cases; without knowledge of the reduced concentration, two patients with substantially reduced citalopram concentration reported that the dose had been doubled after the surgery because of deteriorated mental health. Both had a very low dose of citalopram. Given the dose–response relationship shown for this substance [18], it may be speculated that patients on low doses may be more vulnerable to concentration changes. However, it cannot be excluded that exacerbated psychiatric symptoms in these cases may represent a natural course of the disease rather than being drug-related. Regarding duloxetine, it can be speculated that the reduced dose-adjusted concentration after bariatric surgery is related to a reduced absorption because of insufficient intestinal dwell time of the slow release capsule generally used.

To briefly investigate the generalizability over the drug classes regarding the correlation between logD and the concentration change over the low-calorie diet, we searched the literature for highly hydrophilic and lipophilic substances. However, no conclusions could be drawn from most reports as the temporal relationship between the pre-surgery (baseline) sampling and a potential pre-surgery low-calorie diet was not revealed. In addition, post-surgery values were typically sampled a long time after surgery, making effects of the low-calorie diet and the surgery hard to separate. To circumvent this, results for extremely hydrophilic (logD <  −1.5) or extremely lipophilic (logD > 4) substances, which theoretically can be expected to have a more long-lasting diet effect, were extracted from studies where historical data or matched non-surgery subjects rather than pre-surgery baseline values served as controls. In line with our reasoning of an effect of the low-calorie diet, the extremely hydrophilic substances metformin and moxifloxacin appeared to increase after the bariatric surgery procedure [19, 20], whereas the extremely lipophilic substances tamoxifen, tacrolimus, and sirolimus appeared to decrease [21, 22]. Only two reports were found regarding the correlation between the low-calorie diet per se and drug concentration change, and none of these included highly hydrophilic or lipophilic substances [23, 24]. Taken together, this brief literature review confirms that the present study is the first one to reveal an effect of the pre-surgery, low-calorie diet on drug concentrations, and hence the need for further studies to address the generalizability of the association between logD and concentration changes over a low-calorie diet.

As observed previously in twelve patients on various SSRIs and SNRIs [7], the dose-adjusted concentration of most drugs recovers towards baseline after bariatric surgery. Underlying mechanisms may include that the decrease in glomerular hyperfiltration is fastest at the beginning of a weight reduction regimen [14]. Further, the decrease in liver steatosis may be faster over a 4-week low-calorie diet than during the first month after gastric bypass [17, 25]. Recovered elimination of hydrophilic drugs could perhaps be explained by reduced body water relative to glomerular filtration rate after further weight loss. Increased concentrations of CYP3A substrates, and possibly other phase I-metabolized substances, could depend on decreased pre-systemic metabolism due to the surgical bypass of the most enzyme-rich proximal parts of the small intestine, counteracting possibly increased liver metabolism [26]. However, post-surgery recovery was not always immediate, and no differences between gastric bypass and sleeve gastrectomy were found. This could suggest that other mechanisms are more important, such as adaptive processes in the mucosa, increasing its drug absorption capacity, or a decreased volume of distribution of lipophilic substances at this advanced stage of adipose tissue loss. The distinctive behaviour of fluoxetine might, in addition, be attributable to its saturable kinetics [27, 28], which could render it more sensitive to bypassing of metabolic enzymes. Possibly, the single paroxetine patient could reflect the same mechanism [27, 28].

Strengths of the present study are the large number of patients included, the number of substances covered, the longitudinal design, and the naturalistic setting. Indeed, as far as we are aware, this is the hitherto largest study performed. The longitudinal design, making intra-individual comparisons over time, can reveal causal relationships. Further, the naturalistic setting and consecutive patient recruitment increase the generalizability of the results. Another strength is that we collected samples both before and after the pre-surgery diet, which enabled us to reveal the diet effect related to the lipophilicity of the substances. Through mechanistic reasoning and supported by available literature, these results may be generally applicable.

Limitations of the study include its descriptive nature and reliance on patients’ reporting, for instance regarding dosing and compliance. In addition, few individuals contributed data for substances rarely used by bariatric surgery patients. Nevertheless, these case reports support the general finding regarding drug lipophilicity and drug concentrations following the low-calorie diet, and they may also be valuable in clinical decision-making in the absence of other evidence. The use of dose-adjusted concentrations for comparisons may be regarded as a limitation; although the relationship between dose and concentration has been described as linear for most neuropsychiatric substances [29], exceptions exist [30]. Furthermore, interactions affecting the relationship between the dose and the concentration cannot be excluded. However, this study reveals a conspicuous linear relationship related to the drug lipophilicity and the low-calorie diet, and this finding is not likely to be entirely explained by such interaction effects. Results for specific drugs, though, should be interpreted with caution, rather elucidating the expected direction of the concentration change than providing quantitative estimates.

## Conclusions

Following psychotropic and antiepileptic drug concentrations at the individual level over time before and after bariatric surgery, this study reveals the following novel insights: (i) at the overall level, some reduction in dose-adjusted concentrations can be expected for common antidepressant substances, occasionally associated with contemporary treatment adjustments; (ii) the inter- and intra-individual variations in concentrations are practically unpredictable at the individual level, illustrated by a lack of covariation with individual characteristics in this large data set; (iii) the low-calorie diet drastically affects dose-adjusted serum concentrations of many drugs, often more than the surgery itself; and (iv) the low-calorie diet decreases the concentration of highly lipophilic drugs (such as sertraline and mirtazapine) and increases the concentration of highly hydrophilic drugs (such as pregabalin), suggesting, for the first time, a systematic association between drug properties and concentration changes possibly generalizable to other drug classes. These results imply a need in most patients for longitudinal monitoring of serum concentrations over the low-calorie diet period and up to 12 months after surgery. Post-diet monitoring, perhaps compared to an ideal baseline, pre-diet value, may be particularly relevant to optimizing treatment before the potentially stressful surgery. The systematic effects of the low-calorie diet related to the lipophilicity of the drugs constitute a basis for further research on generalizability and mechanisms.

## Data Availability

The data sets generated and analysed during the current study are not publicly available because of Swedish data protection laws. The data can be shared with authorised persons after approval from the Swedish Ethical Review Authority (https://etikprovningsmyndigheten.se).

## Declarations

### Disclaimer

The funder had no role in study design, data collection and analysis, decision to publish, or preparation of the manuscript.
